# Viral vectors expressing group B meningococcal outer membrane proteins induce strong antibody responses but fail to induce functional bactericidal activity

**DOI:** 10.1016/j.jinf.2022.02.032

**Published:** 2022-03-01

**Authors:** Leanne Marsay, Christina Dold, Gavin K. Paterson, Yuko Yamaguchi, Jeremy P. Derrick, Hannah Chan, Ian M. Feavers, Martin C.J. Maiden, David Wyllie, Adrian V. Hill, Andrew J. Pollard, Christine S. Rollier

**Affiliations:** a Oxford Vaccine Group, Department of Paediatrics, University of Oxford and the NIHR Oxford Biomedical Research Centre, CCVTM, Churchill Lane, Oxford OX3 7LE, United Kingdom; b Jenner Institute, University of Oxford, Old Road Campus Research Building, OX3 7DQ, United Kingdom; c Lydia Becker Institute of Immunology and Inflammation, School of Biological Sciences, Faculty of Biology, Medicine and Health, Manchester Academic Health Science Centre, University of Manchester, Manchester M13 9PL, United Kingdom; d National Institute for Biological Standards and Control, Blanche Lane, South Mimms, Potters Bar, Hertfordshire, United Kingdom; e Department of Zoology, University of Oxford, 11a Mansfield Road, Oxford OX1 3SZ, United Kingdom; f Section of Immunology, Department of Biochemical sciences, School of Biosciences & Medicine, Faculty of Health and Medical Sciences, University of Surrey, Dorothy Hodgkin Building (AY), Guildford, Surrey GU2 7XH, United Kingdom

**Keywords:** Adenovirus, Vector, Vaccine, Outer membrane protein, Meningococcus, Meningococcal disease, Porin

## Abstract

**Objective:**

Adenoviral vectored vaccines, with the appropriate gene insert, induce cellular and antibody responses against viruses, parasites and intracellular pathogens such as *Mycobacterium tuberculosis*. Here we explored their capacity to induce functional antibody responses to meningococcal transmembrane outer membrane proteins.

**Methods:**

Vectors expressing porin A and ferric enterobactin receptor A antigens were generated, and their immunogenicity assessed in mice using binding and bactericidal assays.

**Results:**

The viral vectors expressed the bacterial proteins in an *in vitro* cell-infection assay and, after immunisation of mice, induced higher titres (> 10^5^ end-point titre) and longer lasting (> 32 weeks) transgene-specific antibody responses *in vivo* than did outer membrane vesicles containing the same antigens. However, bactericidal antibodies, which are the primary surrogate of protection against meningococcus, were undetectable, despite different designs to support the presentation of the protective B-cell epitopes.

**Conclusion:**

These results demonstrate that, while the transmembrane bacterial proteins expressed by the viral vector induced strong and persistent antigen-specific antibodies, this platform failed to induce bactericidal antibodies. The results suggest that conformation or post-translational modifications of bacterial outer membrane antigens produced in eukaryote cells might not result in presentation of the necessary epitopes for induction of functional antibodies.

## Introduction

Replication-deficient recombinant adenoviruses can be used to deliver vaccine antigens.^1^ They were originally developed because of their well-recognised ability to induce potent cellular immunity, but a single dose of such vectors has been shown to induce very potent functional antibodies against the encoded gene product (transgene) rapidly (within a week). This was initially demonstrated with vaccines designed against rabies virus^2^ and confirmed with other pathogens including *Plasmodium falciparum*^3^ and Ebola virus.^4^ Subsequently, this technology has been used to develop novel vaccines against various pathogens in both humans and animals,^3–6^ including SARS-CoV-2.^7^ Proteins from viruses and the parasites that cause malaria are naturally produced by eukaryotic cells, therefore, providing the signal sequences are functionally preserved, their expression from a viral vector is likely similar to the natural expression. However, differences between prokaryotic and eukaryotic expression systems may result in a lack of expression of bacterial antigens in mammalian cells, or the loss of protective epitopes, due to misfolding or aberrant post-transcriptional modifications. Despite this, there is evidence that viral vectors can deliver mammalian codon-optimised bacterial antigens that induce functional antibody responses, as seen following immunisation with vectors encoding the secreted proteins *tetanus toxin fragment C* and *protective antigen*, that protect mice against tetanus and anthrax challenge respectively.^8,9^

For some bacterial pathogens, such as the encapsulated bacterium *Neisseria meningitidis*, for which there is a need for an improved vaccines to cover capsular group B (MenB) organisms,^10,11^ the most promising candidate antigens are outer membrane proteins (OMPs). While there are low rates of disease currently, low rates post pandemic have been replaced by some resurgence since reductions in social distancing especially in university students. Moreover, MenB disease is cyclical and thus rates may change in the future. One such MenB OMP is Porin A (PorA), which is an immunodominant protein that induces bactericidal antibodies. Serum bactericidal activity has been associated with protection against meningococcal disease, as demonstrated in humans with antibodies against capsular polysaccharides.^12^ PorA adopts a classic porin *β*-barrel fold. It is predicted to span the outer membrane 16 times, based on the crystal structure of the closely related porin, PorB.^13^ The ends of the *β*-strands are joined by surface-exposed loop regions, two of which, in particular, are subject to sequence hyper-variability (variable regions or VRs),^14^ and are targets for bactericidal antibodies.^15,16^ The ferric enterobactin receptor A (FetA) is an integral OMP, which acts as a TonB-coupled iron transporter, although its precise specificity is unclear.^17–19^ The crystal structure of FetA revealed the canonical 22-stranded *β*-barrel, with a central N-terminal plug domain in the centre, characteristic of this group of outer membrane transporters.^20^ The sequence hypervariability of FetA is concentrated on a surface-exposed sub-domain, consisting of an *α*-helix and return strand.^20^ Native FetA induces bactericidal antibodies in laboratory animals,^21,22^ and humans.^23,24^

PorA and FetA are naturally expressed in outer membrane vesicles (OMVs) derived from bacterial membranes.^25^ Gram-negative OMPs may interact with other outer membrane components such as lipooligosaccharides (LOS) and other outer membrane proteins, which cannot be mimicked by eukaryotic cell membranes. However, DNA vaccines encoding OMPs of *Pseudomonas aeruginosa* and *Borrelia burgdorferi* induce protective antibody responses in mice against these pathogens.^26,27^ Further, a DNA vaccine coding for a PorA epitope has been shown to induce bactericidal antibodies,^28^ indicating that delivery of OMPs by adenoviral vaccines might also result in protective antibody responses. The present study investigated the suitability of adenoviruses encoding bacterial surface proteins to induce antibody responses to *N. meningitidis* PorA and FetA.

## Results

### Full-length PorA and FetA proteins are expressed from DNA and adenoviral vectors

**Fig. 1:**
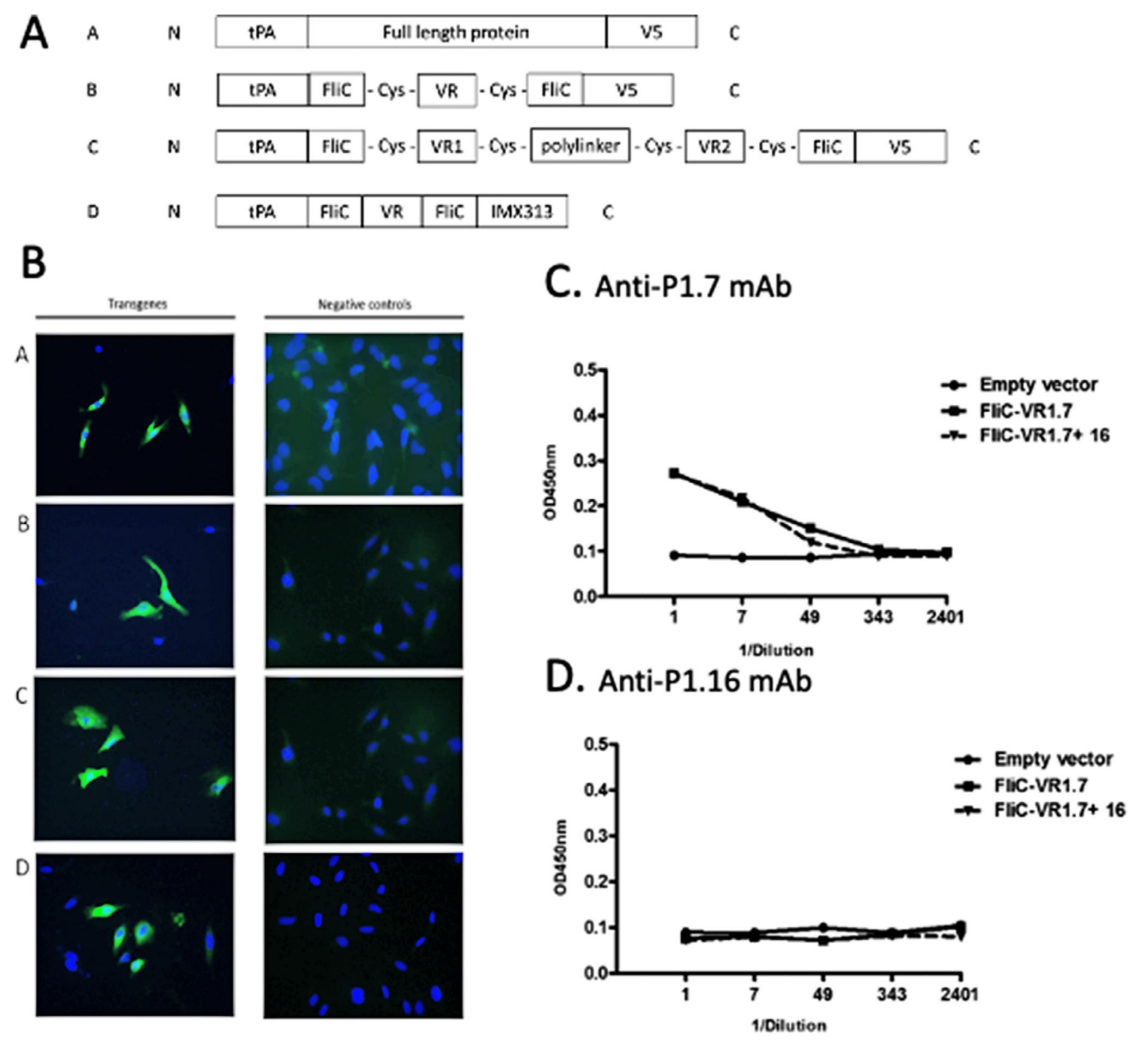
Transgene designs and antigen expression. (A) Schematic diagrams representing the different types of constructs generated for PorA or FetA antigens. For full-length constructs, full-length genes were inserted in frame after the tPA leader, and a V5 marker peptide was added at the C-terminus (Design A). A single PorA VR loop was fused to a FliC scaffold (with or without flanking cysteines), with a V5 peptide sequence at the C-terminus (Design B). Two PorA VR loops were inserted into the FliC scaffold with a flexible polylinker sequence, SGMPGSGPAY, between the VR regions (Design C). A molecular adjuvant (IMX313) was added at the C-terminus (Design D). (B) Detection of PorA and FetA antigens expressed in mammalian cells. HeLa cells were infected with Ad-P1.7,16 and the resulting protein detected using anti-P1.16 mAb (row A), or cells were transfected with plasmids coding for full length FetA3–3 and proteins detected with anti-FetA mouse serum (row B), or FliC-VR3–3 and proteins detected with anti-FetA mouse serum (row C), or FliC-VR1.7-C and protein detected with anti-P1.7 mAb (row D). Negative controls (right image of each row) were stained identically to the test cells (left image each panel). Proteins were detected using a fluorescently labelled secondary antibody (green). Nuclei were counterstained using DAPI (blue). (C and D) Detection of PorA and FetA epitopes in supernatants of cells transfected with FliC-VR constructs. Supernatants from individual transfection supernatants were serially diluted 1:7 in blocking buffer in duplicate. Proteins were detected with anti-P1.7 mAb (C), or anti-P1.16 mAb (D). Plasmids used for transfections are indicated in the figure legends.

Several designs of the bacterial OMPs PorA (types P1.7,16 and P1.7-2,4) and FetA (F3-3) including full length proteins, variable region (VR) loops within FliC scaffolds, human tissue plasminogen activator (tPA) leader fused and non-tPA leader fused (cytosolic) were generated to assess immunogenicity when delivered by adenoviral vectors (Fig. 1A and Table S1). Full length PorA P1.7,16 and FetA F3-3 proteins were detected in HeLa cells by specific antibodies (Fig. 1B). The VR1.16 PorA epitope within PorA type P1.7,16 contained an N-linked glycosylation motif (NLT); to prevent its glycosylation during transit through the Golgi apparatus, full length P1.7,16 without a tPA leader (Cytosolic-P1.7,16) was created. Expression of this transgene was also detected by immunofluorescence (IFA) in HeLa cells using anti-P1.7 mAb targeting the VR1.7 region and bactericidal epitope (data not shown).

**Fig. 2:**
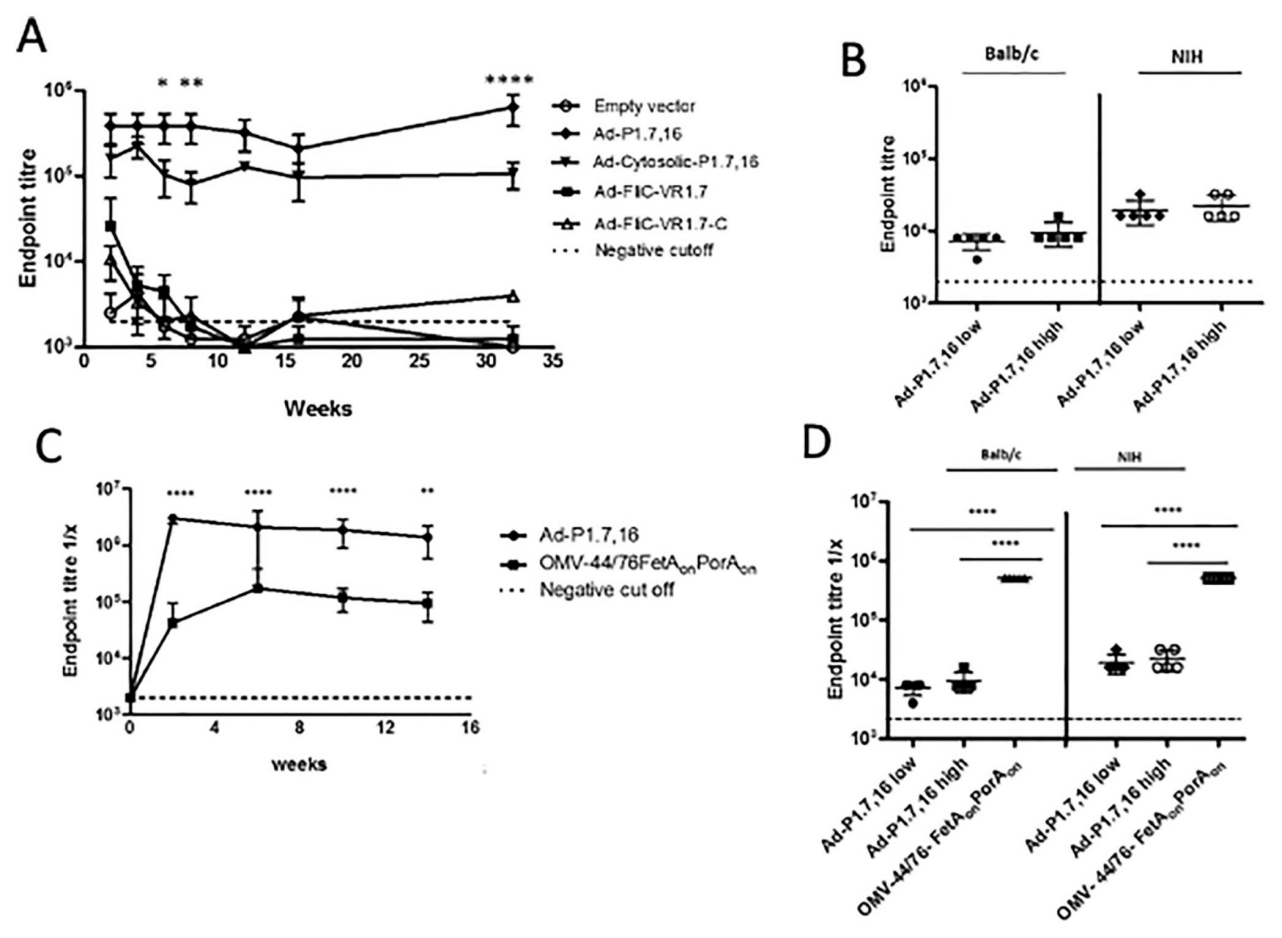
Antibody responses against recombinant PorA (rPorA, P1.7,16). Groups of 4 to 8 BALB/c mice were immunized with a single dose of 10^9^ infectious units (IU) of Ad-vectors and individual serum antibodies against 44/76-SL whole cells measured over time (A). BALB/c and NIH mice (*N* = 5) were immunized with 10^8^ IU (Low) or 10^9^ IU (high) of Ad-P1.7,16 and individual serum antibodies measured 2 weeks post vaccination (B). Comparison of antibody responses induced by Ad-P1.7,16 versus an OMV vaccine (C and D). Individual serum antibody endpoint titres were detected against rPorA P1.7,16 in groups of 4 to 8 NIH mice after a single dose of (10^9^ IU) of Ad-P1.7,16 or 2 doses of 2.5 μg of OMV (44/76-FetA_on_ PorA_on_) for up to 14 weeks (C). Individual serum antibody titers against 44/76-SL whole cells 2 weeks post vaccination of BALB/c and NIH mice (*N* = 5) with 10^8^ IU (Low) or 10^9^ IU (high) Ad-P1.7,16 or 2.5 μg of OMV (44/76-FetA_on_ PorA_on_) (D). In panel C, the response against recombinant PorA protein is shown: A single dose of Ad PorA induces higher antibody titers than OMVs against PorA. In panel D, the response is measured against whole cells, containing other antigens than PorA, several also present in the OMVs. Mean serum individual values with SDs over time (A and C) or individual values at 2 weeks (B and D) are displayed. Dashed lines indicate negative cut-off values (titres obtained with sera from naïve mice). ANOVA was performed with Bonferroni multiple comparisons, ^∗^ *P <* .05, ^∗∗^ *P <* .01 and ^∗∗∗∗^ *P <* .0001 respectively.

It was unknown whether these full-length FetA or PorA would adopt a conformation that could generate bactericidal antibodies. Therefore the variable regions of PorA (VR1.7 and VR1.16) and FetA (VR3-3), representing the known bactericidal epitopes, were inserted into a flagellin (FliC) scaffold derived from *E. coli* flagellin, to induce a conformational constraint of the loops as described earlier^29^ (constructs FliC-VR1.7, FliC-VR1.16 and FliC-VR3-3 respectively, Fig. 1). Expression of FliC-VR1.7, FliC-VR1.16 and FliC-VR3-3 was confirmed by IFA in HeLa cells using PorA- and FetA-specific antibodies respectively (data not shown and Fig. 2C respectively). To further constrain the conformation of the loops, the addition of cysteine residues flanking the FliC-VR1.7 was used to induce disulphide bonds (FliC-VR1.7-C). This has previously been shown to improve immunogenicity of PorA peptides.^29^ Expression of FliC-VR1.7-C was confirmed using anti-P1.7 mAb in HeLa cells by IFA (Fig. 2D), indicating that the additional cysteines did not disrupt binding of anti-P1.7 mAb to the VR1.7 epitope. The FliC scaffold was also used to display the VR1.7 and VR1.16 together, both with and without flanking cysteines (FliC-VR1.7 + 16 and FliCVR1.7 + 16-C respectively). Simultaneous expression of both the VR1.7 and VR1.16 was confirmed by IFA in HeLa cells using anti-P1.7 and anti-P1.16 mAbs (Fig. S1).

The multimerization tag IMX313 has previously been shown to enhance the immune response to adenoviral vectored antigens.^3^ Therefore, a vaccine containing the FliC-VR1.7 fused to the IMX313 molecular adjuvant (FliC-VR1.7-IMX313) was generated. Expression of FliC-VR1.7-IMX313 was confirmed by IFA in HeLa cells using anti-P1.7 mAb (data not shown).

**Fig. 3:**
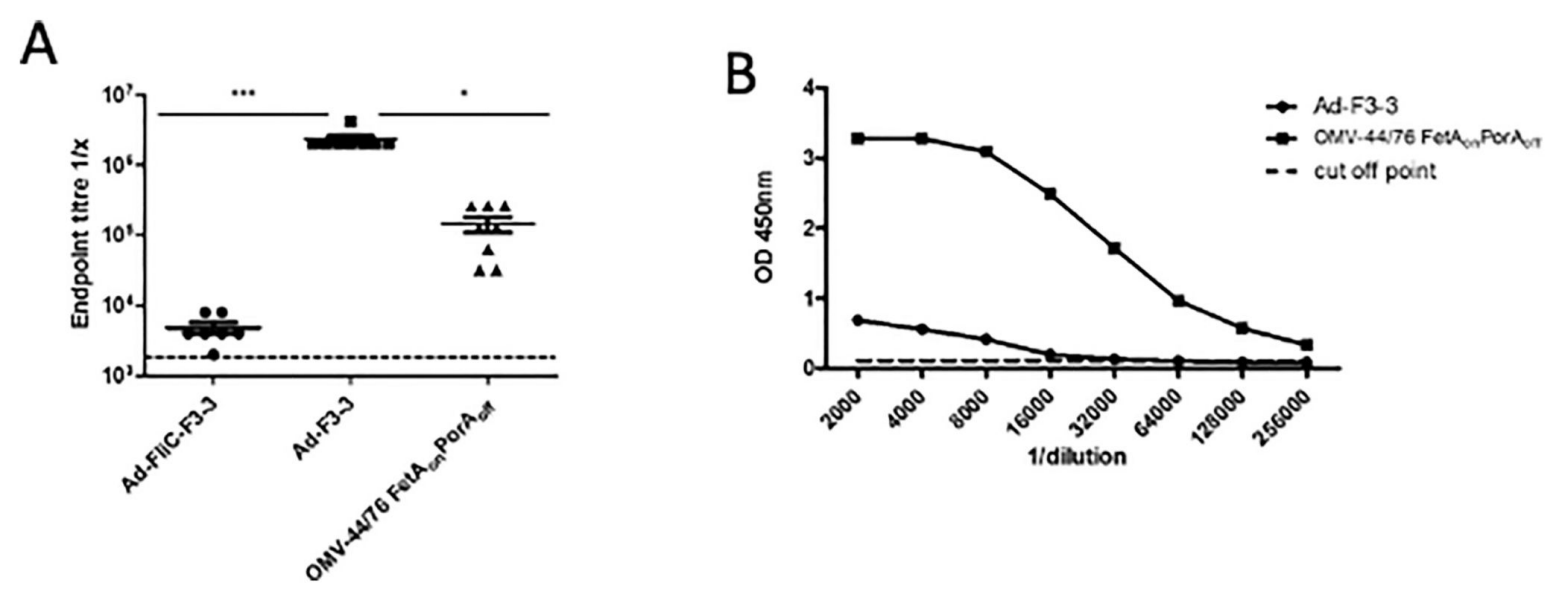
Comparison of antibody responses induced by Ad-F3–3 vaccines and OMV vaccine. BALB/c mice received 10^9^ IU of full length Ad-F3–3 (*N* = 7), Ad-FliC-VR3–3 (*N* = 8) or 5 μg of OMV (44/76-FetA_on_ PorA_off_, *N* = 8). Individual antibody responses against recombinant rF3–3 (A) or against 44/76-FetA_on_PorA_off_ whole cells (B, pooled sera from each group) 6 weeks post vaccination are shown. Groups of 7 or 8 mice were used for the experiments as indicated above. Kruskal-Wallis test with Dunn’s multiple comparison was used to perform comparisons between the groups ^∗^ *P* ≤ 0.05 and ^∗∗∗^ *P* ≤ 0.001.

The binding of anti-P1.16 mAb to both the full length PorA 1.7,16 and the PorA VR only transgene proteins FliC-VR1.16 and FliC-VR1.7-VR1.16 in the IFA was unexpected due to the predicted N-linked glycosylation site within the VR1.16 epitope. Post-translational glycosylation of the PorA VR1.16 was expected to block anti-P1.16 antibody binding. One possible explanation for the positive IFA results is that the anti-P1.16 mAbs were binding to the transgene proteins in the cytosol before they underwent post-translational glycosylation. Indirect ELISA was therefore performed to detect the presence of secreted VR1.7 and VR1.16 epitopes in culture supernatants of HeLa cells transfected with plasmids coding for FliC-VR1.7, FliC-VR1.16 and FliCVR1.7-VR1.16. While FliC-VR1.7 and FliC-VR1.7-VR1.16 were detected with anti-P1.7 mAb (Fig. 3A), FliC-VR1.16 and FliC-VR1.7-VR1.16 were not detected by anti-P1.16 mAb (Fig. 3B). These findings suggested that the VR1.16 epitope may not have adopted the correct conformation or may have been altered during post-translational modification within the HeLa cells.

### PorA P1.7,16 expressing vectors induce antibody responses in mice

The PorA P1.7,16 vaccines Ad-P1.7,16, Ad-cytosolic-P1.7,16, Ad-FliC-VR1.7 and Ad-FliC-VR1.7-C were assessed for immunogenicity in mice. Ad expressing unmodified FliC was used as negative control. Specific antibody responses were elicited by the full-length Ad-P1.7,16 and Ad-cytosolic-P1.7,16 vectors, as early as two weeks after a single vaccination, and were maintained for up to 32 weeks Fig. 2A). In accordance with previous findings, immunisation with Ad-P1.7,16 with the tPA leader induced consistently higher antibody titres than the Ad-cytosolic-P1.7,16 without the tPA leader. The difference was significant at 6, 8, and 32 weeks after vaccination (*P <* .05, *P <* .01 and *P <* .001 respectively). In comparison, the Ad-FliC-VR1.7 and Ad-FliC-VR1.7-C vectors, which only expressed the VR1.7 epitope, elicited low antibody titres, only significantly higher in the Ad-VR1.7 group as compared with the Ad-FliC-control group two weeks post-vaccination (*P <* .001, Fig. 2A). The addition of flanking cysteines at the base of the 1.7 epitope in the Ad-FliC-VR1.7-C vaccine did not enhance antibody titres. To confirm that antibodies generated by immunisation with Ad-P1.7,16 could bind PorA P1.7,16 naturally expressed by meningococci, ELISA against whole cell preparations of strain 44/76-SL were performed. BALB/c and NIH mice immunised with a low (10^8^ infectious units, IU) or high (10^9^ IU) dose of Ad-P1.7,16 elicited detectable antibody responses against the whole bacterial cells two weeks post vaccination (Fig. 2B).

The immunogenicity of Ad-P1.7,16 was compared to that of an outer membrane vesicle (OMV) vaccine (44/76-FetA_on_PorA_on_), expressing known amounts of PorA and FetA.^24^ While it is not possible to know the exact amount of PorA delivered by the Ad vector, as compared with the one in the OMV preparation, using an OMV is the optimal formulation that successfully induces PorA-specific bactericidal responses.^30^ PorA-specific antibody levels induced by Ad-P1.7,16 were higher than those induced by an OMV vaccine when measured against rPorA P1.7,16 protein (Fig. 2C). When whole cells (44/76-SL) were used as target in the ELISA assay, greater levels of antibodies were detected in response to the OMVs compared with Ad-P1.7,16 (Fig. 2D), as expected since OMV vaccines induce antibodies against several other antigens.

### FetA F3-3 expressing vectors induce antibody responses in mice

FetA F3-3 expressing vectors Ad-F3-3 (full length) and Ad-FliC-VR3-3 (VR3-3 only) elicited FetA-specific antibody responses in mice as measured against rF3-3 protein (Fig. 3A). An OMV comparator that did not contain the immunodominant PorA but had a defined quantity of FetA (7.8% of total OMPs), was used to assess the FetA response to an OMV vaccine (OMV-44/76FetA_on_PorAoff).^24^ Immunisation with Ad-F3-3 resulted in significantly higher antibody titres compared with the OMV comparator and Ad-FliC-VR3-3 immunised mice 6 weeks post immunisation (*P* ≤ 0.05 and *P* ≤ 0.001, respectively). Mice in the OMV comparator group elicited F3-3 specific antibody titres that were not significantly greater as compared with those elicited by Ad-FliC-VR3-3-immunization. Ad-F3-3 induced anti-FetA antibodies bound naturally expressed FetA F3-3 present in whole cell preparations (Fig. 3B). As with the P1.7,16 adenoviral vaccines, greater levels of antibodies were detected in response to the OMVs when whole bacterial cells were used for antibody capture, likely due to induction of antibodies against multiple bacterial antigens in the OMV vaccine.

### Vector-induced anti-PorA and FetA antibodies are not bactericidal

The functional capacity of the antibodies induced by the vectored vaccines was measured by serum bactericidal activity (SBA). None of the viral vectors induced a detectable bactericidal response, despite the particularly high antibody concentrations detected after immunisation with the full-length vaccines (Table 1). Antibody subclass ELISAs were performed on sera taken from mice immunised with a single dose of 10^9^ IU of Ad-P1.7,16 or Ad-FliC-VR1.7 or two doses of 5 μg of OMV comparator (44/76-FetA_*on*_PorA_*on*_) against rPorA P1.7,16. The levels of IgG3 antibodies were low or non-detectable in all vaccine groups (data not shown). Sera from Ad-P1.7,16 and OMV immunized mice had detectable levels of IgG1, IgG2a and IgG2b (Fig. S2), whereas mice immunised with Ad-FliC-VR1.7 only elicited IgG2a antibody titres above the negative cut-off. The antibody endpoint-titres were reduced across all three subclasses when 1M sodium thiocyanate was included in the diluent (only significant in the OMV immunized mouse sera for IgG2b antibodies, *P* ≤ 0.01, data not shown).

### Effect of boosting adenoviral vector induced antibody responses with OMV vaccines

**Fig. 4:**
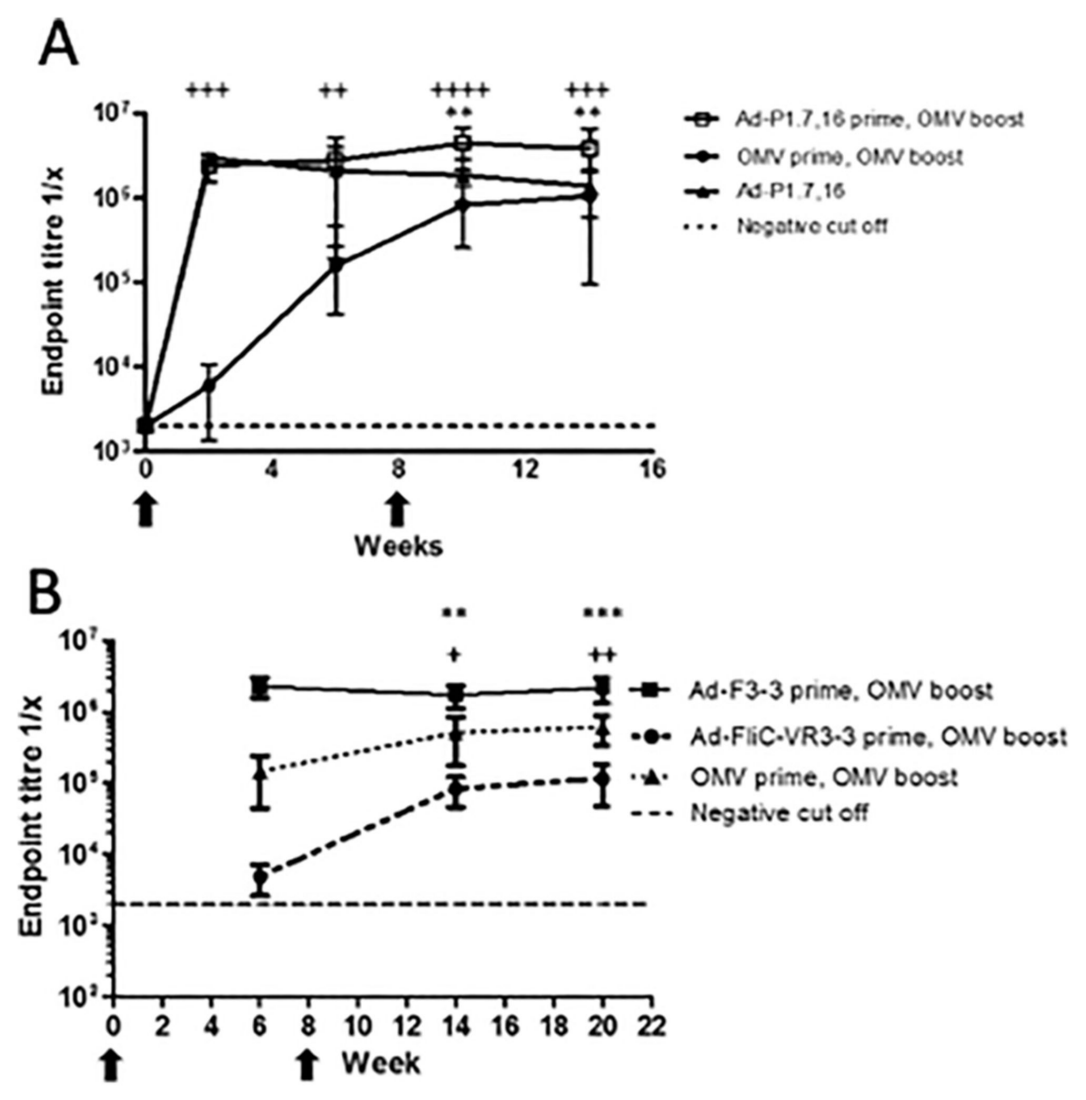
Antibody responses after Ad prime and OMV boost. Groups of 5 NIH mice were immunised with 10^9^ IU of Ad-P1.7,16 or 2.5 μg of OMVs (44/76 FetA_*on*_ PorA_*on*_) according to the figure legend (A). Immunisation times are marked with arrows. Antibody responses were measured by ELISA against recombinant PorA rP1.7,16. Mean values with SD are displayed. Asterisks denote two-way ANOVA comparisons with Bonferroni multiple testing between groups Ad-P1.7,16-prime, OMV-boost and Ad-P1.7,16 alone. Crosses denote two-way ANOVA comparisons between the groups Ad-P1.7,16-prime, OMV-boost and OMV-prime, OMV-boost. ∗∗ or ++ = *P* ≤ 0.01, +++ = *P* ≤ 0.001 and ++++ = *P* ≤ 0.0001. Antibody responses to FetA F3–3 (B) Groups of 8 BALB/c mice were immunised with 10^9^ IU of adenoviral vectors Ad-F3–3, Ad-FliC-VR3–3 or 5 μg OMVs (44/76 FetA_*on*_ PorA_*off*_), and then boosted with OMV-44/76 FetA_*on*_ PorA_*off*_ at week 8, vaccination time points are marked with arrows. ELISAs were performed against recombinant FetA rF3–3. Kruskal Wallis with Dunn’s multiple comparison tests were used to perform comparisons between week 6 vs. week 14 or week 20 for Ad-FliC-VR3–3 boosted with OMV (asterisks) or OMV boosted with OMV (crosses) + = *P* ≤ 0.05, ^∗∗^ or ++ = *P* ≤ 0.01 and ^∗∗∗^ = *P* ≤ 0.001

The use of adenoviral vaccines to prime a strong antibody response after a heterologous protein boost has been described previously.^31–33^ If the adenoviral vectors induced low levels of bactericidal antibodies directed against the protective VR regions of PorA P1.7,16, this antibody response might be boosted by an OMV vaccine that displays the VR regions on the surface of OMVs. This would result in greater SBA titres after heterologous Ad-prime-OMV-boost than is seen for OMV vaccines alone. To address this question, groups of NIH mice were immunised with 10^9^ IU of the viral vector Ad-P1.7,16 with or without a boost of 2.5 μg of OMVs (44/76 FetA_*on*_PorA_*on*_). PorA P1.7,16 specific antibody levels were significantly higher in Ad-P1.7,16 primed - OMV boost (containing the homologous PorA subtype) (Ad-P1.7,16-prime, OMV-boost) compared with Ad-P1.7,16 immunised mice that did not receive an OMV boost (Ad-P1.7,16, *P* ≤ 0.01) (Fig. 4A). The Ad-P1.7,16 prime-OMV-boost group also had higher levels of P1.7,16-specific antibodies compared with the OMV-prime-OMV-boost comparator group at all time-points pre- and post-boost (*P* ≤ 0.01 at week 6, *P* ≤ 0.001 at weeks 2 and 14 and *P* ≤ 0.0001 at week 10).

The bactericidal responses induced by an Ad-P1.7,16 prime followed by an OMV (44/76 FetA_*on*_PorA_*on*_) boost were assessed using human complement. The Ad-P1.7,16 prime did not induce hSBA responses above that of naïve mice (1:4), whereas mice immunized with the OMVs elicited a hSBA titre of 1:64 two weeks after the primary vaccination (Table 2). SBA responses after an OMV boost were 1:128 and 1:1024 for the Ad-P1.7,16 and OMV primed groups respectively, indicating that Ad-P1.7,16 was not able to efficiently prime a bactericidal antibody response that could be boosted by an homologous OMV vaccine.

Similarly, the effect of boosting anti-F3-3 antibodies with an OMV vaccine was also assessed. Boosting of anti-F3-3 antibodies induced by Ad-F3-3, Ad-FliC-VR3-3 and the comparator OMV (44/76 FetA_*on*_PorA*off*) vaccine by subsequent immunisation with OMVs (44/76 FetA_*on*_PorA*off*) at week 8 was measured by ELISA against the recombinant protein. Boosting Ad-F3-3 (full length) with OMVs did not result in higher endpoint titres compared to the titers prior to the boost (Fig. 4B). Boosting with OMVs resulted in higher antibody end-point titres in the Ad-FliC-VR3-3 (F3-3 epitope) and OMV primed mice (week 6 vs. week 14, *P* ≤ 0.01 and *P* ≤ 0.05 respectively), which were maintained until week 20 (week 6 vs. week 20, *P* ≤ 0.001 and *P* ≤ 0.01 respectively). However, only the OMV prime-boost vaccine regimen elicited a detectable rSBA titre of 1:32, six weeks after boost (Table 3).

### PorA subtype P1.7-2,4 expressing adenoviral vaccines also induce strong antibody responses but no bactericidal activity

**Fig. 5:**
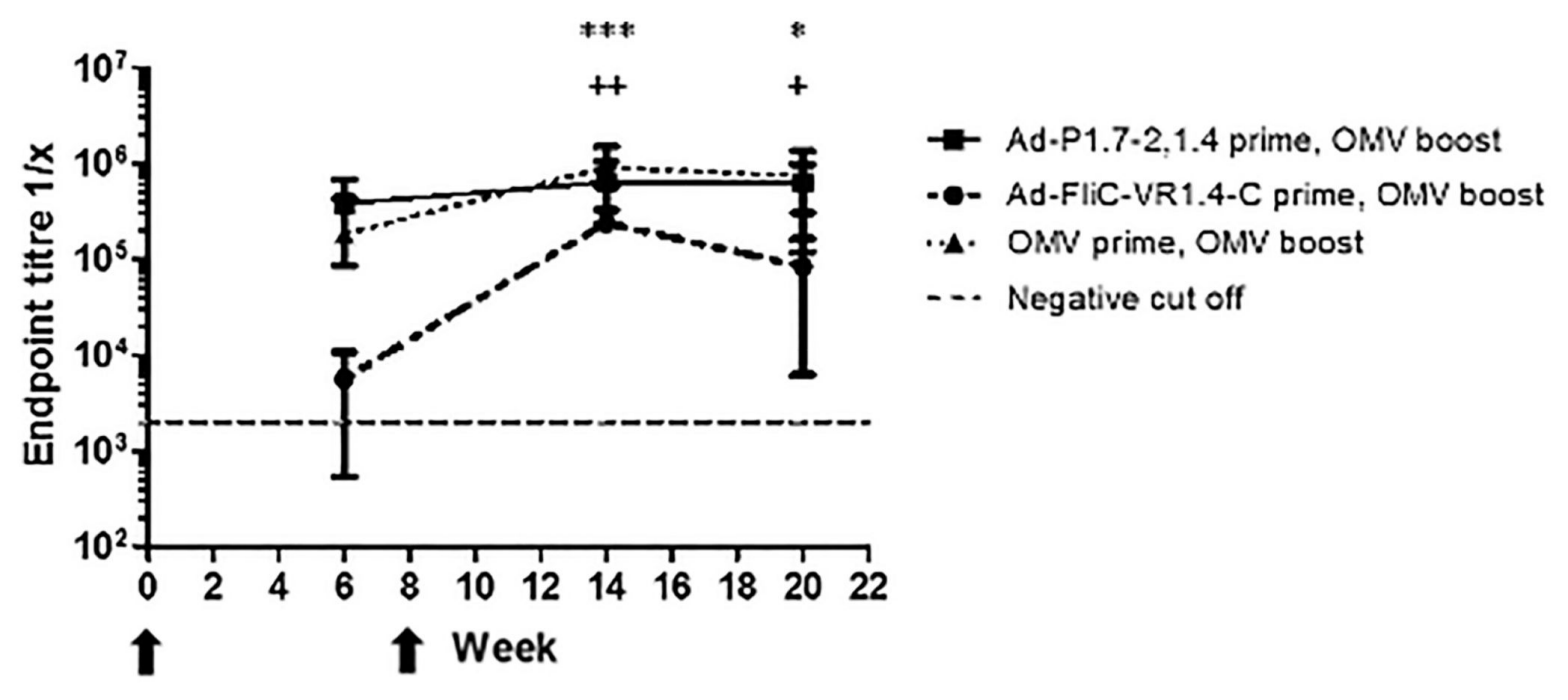
Antibody responses to PorA P1.7–2,4 adenoviral vectors with OMV boosting. Groups of 8 BALB/c mice were immunised with 10^9^ IU of adenoviral vectors Ad-P1.7–2,4, Ad-FliC-VR1.4-C or 5 μg of OMVs from strain NZ98/254 and all boosted with OMVs at week 8, Vaccination time-points are marked with arrows. ELISAs were performed against recombinant PorA rP1.7–2,4. Kruskal Wallis with Dunn’s multiple comparison tests were used to perform comparisons between week 6 vs. week 14 or week 20 for Ad-FliC-VR1.4-C boosted with OMV (asterisks) or OMV boosted with OMV (crosses) ^∗^ or + = *P* ≤ 0.05, ++ = *P* ≤ 0.01 and ^∗∗∗^ = *P* ≤ 0.001 .

The absence of bactericidal activity induced by Ad-P1.7,16 could have been due to N-glycosylation of the VR2 epitope (1.16), as there is evidence that the 1.7 epitope is less effective at inducing bactericidal antibodies than P1,16.^34^ Therefore, vectors encoding a different PorA (P1.7-2,4) that does not contain an N-linked glycosylation motif in the VR and protective regions were created. Bactericidal activity against this PorA is primarily to the P1.4 VR2 epitope.^16^ The antibody levels induced by adenoviral vaccines coding for P1.7-2,4 antigens were compared with homologous (PorA subtype) OMVs (NZ98/254). In BALB/c mice, Ad-P1.7-2,4 induced high levels of antibodies detected against recombinant PorA rP1.7-2,4 by ELISA 6 weeks post immunisation (Fig. 5), which were comparable to the levels of P1.7-2,4-specific antibodies elicited in OMV immunised mice. Immunisation with Ad-FliC-VR1.4-C induced detectable but weak antibody responses 6 weeks post immunisation. Boosting with OMVs at week 8 resulted in increased end-point titres in mice primed with Ad-FliC-VR1.4 or OMVs after the booster (week 6 vs. week 14, *P* ≤ 0.001 and *P* ≤ 0.01 respectively; week 6 vs. week 20, *P* ≤ 0.05 for both groups). Mice that received Ad-P1.7-2,4 as the priming vaccine did not elicit significantly higher PorA P1.7-2,4-specific antibody levels following an OMV boost. No hSBA activity was detected 6 weeks after a single immunisation with Ad-P1.7-2,4 or Ad-FliC-VR1.4 (data not shown). Mice immunised with NZ98/254 OMVs elicited hSBA geometric mean titre (GMT) of 17.7 (95% CI 5.1, 61.7) 6 weeks after a single immunisation (Table 4). When Ad-P1.7-2,4 immunized mice were boosted with NZ98/254 OMVs, a hSBA GMT of 58.7 (95% CI 12.3, 280.0) was measured 6 weeks after the OMV boost (Week 14), which was not significantly higher 6 weeks after OMV immunisation alone. Two doses of OMVs as prime and boost immunisations elicited a hSBA GMT of 78.0 (95% CI 48.0, 126.7) 6 weeks after the boost immunisation (week 14) which was significantly higher than 6 weeks after the prime (*P* = .018).

## Discussion

We demonstrate here that adenoviral vectors encoding PorA or FetA meningococcal antigens induce strong and long-lived antibody responses in mice after a single dose. However, no serum bactericidal activity was detected in mice immunised with any of the adenoviral vaccines alone and there was no appreciable increase in bactericidal antibodies in response to combining Adprime with an OMV boost as compared with using OMV only. Several viral vectors have been shown to induce functional antibody responses, against rabies,^35^ RSV,^36^ SARS-CoV-2,^37^ malaria,^38^ or antitumor functions.^39^ However, in these cases the antigens are naturally expressed in eukaryote cells. For bacterial antigens, protective responses have been raised against toxins such as anthrax,^40^ but for most bacterial antigens incorporated into adenovirus vaccines such as *S. pneumoniae, Y pestis* and *S. aureus*, the role of the T-cell response was not analysed separately to the antibody-mediated effect in the protection experiments.^41–43^ Therefore, little is known about induction of functional antibodies to transmembrane bacterial outer membrane proteins engineered in adenovirus vaccines.

There are a number of possible explanations for the failure to generate functional anti-bacterial antibodies. The PorA and FetA proteins, when expressed by mammalian cells, may not have adopted a conformation that would generate bactericidal antibodies. PorA and FetA are transmembrane proteins that form characteristic loops on the meningococcal surface. Their expression and potential post-translational modification in mammalian cells may have resulted in altered structures, which would suggest that vaccines based on gene expression (DNA, viral vectors or mRNA) may not be suitable for this type of bacterial antigens. Moreover, the location of the PorA and FetA antigens expressed by the vectors in mammalian cells remains uncertain: In bacteria there exist multiple systems for protein trafficking and membrane insertion (such as the Bam system,^44^) that are absent in eukaryotic cells. Attempts to identify PorA P1.7,16 localization to either the cytoplasm or cell membrane of Ad-cytosolic-P1.7,16 or Ad-P1.7,16 infected HEK293 cells by western blot of cell fractions was not conclusive (data not shown), probably due to the low amount of protein produced or their instability in cells. The native structure of PorA antigens is known to be important for the induction of bactericidal antibodies against meningococci,^28^ in agreement with current knowledge of the structures of PorA and FetA.^13^ Although the mouse anti-PorA bactericidal antibodies used in this study reacted to linear epitopes, they were elicited using OMVs.^45^ Also, regions of sequence hypervariability in PorA, FetA and similar integral OMPs are frequently referred to as ‘loops’, but such structures do not form in isolation and are adjacent to, and stabilized by, other parts of the protein or other proteins (for example Rmp). Antibody recognition of surface-exposed regions is likely to be mediated by conformational epitopes, although recognition may be mimicked to some extent by short peptides encompassing the main antigenic loop.

Another possible explanation for the lack of bactericidal antibodies is that Ad-PorA and Ad-FetA vaccines did not induce complement fixing subclasses of IgG that are required for bactericidal activity. In mice, the hierarchy for serum bactericidal activity for the P1.16 epitope has been determined as IgG3 *>>* IgG2b > IgG2a *>>* IgG1.^46^ In the present study, neither OMVs nor adenoviral vaccines were effective at inducing IgG3 antibodies, but there was a trend for greater IgG2b responses after OMV immunization compared with the adenoviral vectors, which induced more IgG2a antibodies. The lack of IgG3 response and greater IgG2b response to the OMV vaccine in the present study was also seen for the vaccine VA-MENGOC-BC in mice, with IgG2b antibody titres correlating with SBA activity.^47^ Although lower in quantity, IgG2b antibodies were detected in response to vaccination with Ad-P1.7,16, so the subclass of IgG antibodies alone cannot account for the lack of bactericidal activity in response to this vaccine.

The Ad-cytosolic-P1.7,16 vaccine was less immunogenic than the secreted Ad-P1.7,16 counterpart, which contained a tPA leader sequence known to enhance secretion and presentation of antigens to the immune system.^48^ However, omitting the secretory signal did not result in induction of bactericidal activity, suggesting that N-linked glycosylation, that may happen with secretion of Ad-P1.7,16, was not directly responsible for the lack of bactericidal activity.

Anticipating folding problems with the full-length PorA and FetA proteins when delivered by adenoviral vaccines, we developed VR loop constructs displayed within a flagellin scaffold^49^ to constrain the VR peptides to form loop structures that would more closely mimic the native exposed loops of PorA and FetA. The ELISA antibody titres to the PorA and FetA antigens were lower for the VR-peptide vaccines as compared with their full-length counterparts, because these constructs only contained one epitope. However, the antibody responses were potentially all directed against the bactericidal epitopes^14,16,21,29^ and thus were expected to have high SBA activity. Although the transgene products bound *in vitro* to mAbs known to be bactericidal, they did not elicit a bactericidal antibody response *in vivo*. Conversely, a DNA vaccine for the PorA P1.16-2 VR has been shown to generate bactericidal antibodies, albeit at low titers,^28^ a common problem with peptide or epitope-based vaccines. While incorrect conformation may be the main factor behind the lack of functionality, it may also be due to the lack of interactions with other proteins in the membrane (PorB, Rmp) which form complexes with PorA and contribute to stability.^50^ A recent study used the hepatitis B core protein virus-like particle (HBc) to display two different meningococcal antigens,^51^ factor H binding protein and the adhesin NadA^11^; domains from these two antigens were incorporated into HBc by genetic fusion and expressed in *E. coli*. Antibody responses in mice were elicited against both antigens, but significant SBA titres were only obtained against the NadA component. Structural analysis suggested that the NadA domain was folded, whereas the Factor H binding protein domain was not. These observations concur with those here and emphasize the importance of retaining conformational epitopes in order to induce SBA responses.

In conclusion, PorA and FetA are attractive vaccine candidates for protein-based substitutes for MenB capsular vaccines, but their use is complicated by the fact that they are transmembrane proteins, and therefore need to be solubilised in detergent or, better, reconstituted into liposomes.^52^ The adenoviral vectored platform appears efficient to generate antibodies against these meningococcal OM, but not bactericidal ones; an OMV or other ways to restore conformation to exogenously produced antigens may be required. With only two proteins tested however, other integral membrane proteins or surface lipoprotein antigens may yield more promising results. In a recent phase I trial, an OMV expressing PorA P1.7,16 and a defined quantity of F3-3 (7.7%) induced bactericidal antibody responses to both antigens in humans.^24^ Harnessing the potent antibody and T-cell inducing capacity of viral vectors to allow reduction in dosing schedules and induce more persistent responses than OMVs was attractive, but this delivery system may not be appropriate for the induction of antibodies to conformational epitopes in complex transmembrane bacterial proteins. These results are expected to apply to DNA and mRNA-based platforms. These observations however do not preclude the use of other delivery systems for PorA and FetA, through improved methods that ensure the preservation of conformational epitopes.

## Materials and methods

### Generation of vaccines and recombinant proteins

PorA and FetA sequences with the Genbank accession numbers X52995.1 (strain 44/76), AF226337.1 (NZ98/254) and X89755.1 (44/76) were used (Supplementary Table S1). Where appropriate, N to Q amino acid substitutions were made to remove potential N-linked glycosylation sites. Native sequences were codon optimised for expression in humans (GeneArt, Regensburg, Germany).

Transgenes were cloned into plasmids containing *att*R1 and *att*R2 recombination sites (Gateway® Life technologies, CA, USA) under control of a CMV promotor. The human tissue plasminogen activator (tPA) leader was fused to the transgenes in-frame to promote secretion of the antigens, unless otherwise stated, to improve production and presentation of the antigen to the immune system as previously suggested.^48^ For expression of only the bactericidal epitopes, variable regions from PorA and FetA sequences were fused to replace the central portion (D3) of flagellin protein from *Escherichia coli* as described previously.^49^ Cysteine residues flanking the variable loops were engineered where indicated to further constrain the conformation of the epitopes. Transgenes were recombined with pAd-PL DEST using LR Clonase (Invitrogen) to generate recombinant E1 and E3 deficient human adenovirus serotype 5 (AdHu5). The viruses were produced as previously described.^53^ Viral vectored vaccines were formulated in endotoxin-free PBS. Outer membrane vesicle (OMV) vaccines were produced from the MenB strains NZ98/254, 44/76-FetA_*on*_PorA_*on*_ and 44/76-FetA_*on*_PorA*off* by detergent extraction as previously described^24^; the latter two strains are mutants of 44/76 that were created to assess bactericidal activity against PorA and FetA individually. All OMV vaccines were formulated to contain either 2.5 μg or 5 μg of total protein in 20 mM TRIS buffer pH 7.0–7.5 (Sigma Aldrich, MO, USA) with 85 μg of Alhydrogel™ per dose. Recombinant PorA and FetA (also known as FrpB) were expressed and purified as described by Awanye et al.^54^ and Saleem et al.^20^ Briefly, each integral membrane protein was expressed without signal peptide into inclusion bodies, which were solubilized into chaotrope, refolded by dilution into detergent and subsequently purified by metal chelate affinity and size exclusion chromatography.

### Immunofluorescence assay (IFA)

HeLa cells were seeded into 6 well culture plates containing rat collagen coated coverslips (BD Bioscience, NJ, USA). Cells were transfected with plasmid DNA containing the transgenes (stated in the figure legends) using Lipofectamine 2000 according to the manufacturer’s instructions (Life technologies, CA, USA). Alternatively, HeLa cells were infected with adenoviruses expressing transgenes at a MOI of 100. Transfected and infected HeLa cells were left overnight at 37 °C with 5% CO_2_. Cells were fixed with 4% paraformaldehyde and permeabilised with 0.2% Triton X-100 in PBS. Proteins were detected using an appropriate mouse primary monoclonal antibody (anti-P.1.7 [01/514], anti-P1.16 [01/538], anti-P1.4 [02/148]), or polyclonal antibody (mouse FetA F3-3 immunised sera, NIBSC, UK), followed by goat anti-mouse IgG conjugated to Alexafluor 488 (Life technologies, CA, USA). Cell nuclei were counterstained with DAPI and visualised using a Leica DMI3000 B microscope.

### Immunisation experiments in mice

Procedures were performed according to the U.K. Animals (Scientific Procedures) Act 1986 and were approved by the University of Oxford Animal Care and Ethical Review Committee. Six to 8-week-old female BALB/c-OlaHsd and NIH-OlaHsd mice (Harlan, UK) were housed in specific pathogen-free conditions. Mice were immunized with a single injection of 10^8^ or 10^9^ infectious units of each vaccine. All vaccines were given intramuscularly, with an 8-week interval when boosting was performed. Blood was collected from tail bleeds or terminal cardiac bleeds at various time points and allowed to clot, centrifuged at 15,000 x g for 10 min. Sera were aliquoted and stored at -20 °C until use.

### Detection of anti-PorA or FetA antibodies by ELISA

Immulon 2HB Plates (Thermo Fisher Scientific, MA, USA) were coated with either heat-killed whole cell preparations of *N. meningitidis* in PBS (OD600 0.1), or recombinant PorA or FetA proteins in carbonate bicarbonate buffer (Sigma Aldrich, MO, USA) at 2 μg/ml at 4 °C overnight. Plates were washed with PBS Tween 20 at 0.05% before block with 1% BSA in PBS (all Sigma Aldrich, MO, USA) for 2 h at 37 °C. Sera were diluted 1:2000 in blocking buffer and serially diluted in duplicate at 4 °C overnight. For assessment of relative avidity of antibodies, 1M sodium thiocyanate (Sigma Aldrich, MO, USA) with 0.05% Tween 20 was added to the dilution buffer of samples where stated. HRP-conjugated goat anti-mouse secondary antibody (Jackson ImmunoResearch inc. PA, USA) was added at 1:10,000 (total IgG) or 1:20,000 (IgG1, IgG2a, IgG2b or IgG3 subclass) and incubated for 2 h at 37 °C. Plates were developed with TMB solution (Sigma Aldrich, MO, USA) and stopped with 2M H_2_SO_4_. Endpoint-titres were determined as the reciprocal of the dilution giving an OD450 nm reading above that obtained for naive control wells plus 2xSD of six replicates for each plate.

### Indirect ELISA to detect antigen expression

MaxiSorp ELISA plates (Thermo Fisher Scientific, MA, USA) were coated with anti-V5 tag antibody (Abcam, UK) in PBS (Sigma Aldrich, MO, USA) at 2 μg/ml at 4 °C overnight. Plates were washed with PBS Tween 20 (0.05%) and blocked with 1% BSA in PBS (all Sigma Aldrich, MO, USA). Supernatants from individual transfection supernatants were serially diluted 1:7 in blocking buffer in duplicate and left at 4 °C overnight. Plates were washed before addition of anti-P1.7, anti-P1.16 or anti-F3-3 antibodies (NIBSC, UK) and incubated for 1 h at room temperature (RT). HRP-conjugated goat anti-mouse secondary antibody (Jackson ImmunoResearch inc. PA, USA) was added at 1:10,000 dilution and incubated for 1 h at RT. Plates were developed with TMB solution (Sigma Aldrich, MO, USA) and stopped with 2M H2 SO4.

### Serum bactericidal assay (SBA)

The serum bactericidal assay (SBA) measures complement-dependent bacterial lysis mediated by antibodies and in humans is the correlate of protection used for licensure of meningococcal vaccines. The SBA was performed as described previously.^24^ Exogenous rabbit (rSBA) or human (hSBA) complement with no intrinsic bactericidal activity were used at 25% (vol/vol) against the target bacterial strains. Rabbit complement was used first for screening of the vaccine candidates in order to not waste human complement if results were negative, human complement was used in subsequent experiments. Human complement was sourced from consenting healthy adults. Test sera were heat inactivated at 56 °C for 30 min to remove intrinsic complement activity.^24^ Bacterial strains were grown overnight for single colonies on blood agar plates at 37 °C and 5% CO_2_. Approximately 50 colonies were then sub-cultured for 4 h before being reconstituted in Hanks buffered salt solution (Gibco) with 0.5% bovine serum albumin (Sigma Aldrich, MO, USA). The bacteria were further diluted to give approximately 100 colony forming units per 10 μl used for the assay. A titre was defined as the reciprocal of the highest dilution of serum that yielded ≥50% decrease in colony forming units relative to that of control wells within 60 mins at 37 °C without CO_2_.

### Statistics

Statistical analysis were performed using two-way ANOVA with Bonferroni post-tests, one-way ANOVA with Dunns multiple comparisons test, Mann Whitney U test or Kruskal-Wallis where appropriate and as stated in figure and table legends, using Prism 5 (Graphpad, software Inc. CA, USA). Test were two-tailed *Supplementary materials:* Description of transgene designs, Table S1. Detection of simultaneous expression of VR1.7 and VR1.16, Fig. S1. IgG subclass ELISA with relative avidity in the presence of chaotropic salts for Ad-P1.7,16 and OMV vaccines, Fig. S2.

## Supplementary Material

Supplementary material associated with this article can be found, in the online version, at doi:10.1016/j.jinf.2022.02.032.

Suppl

## Figures and Tables

**Table 1:** Serum bactericidal titres after a single dose of PorA P1.7,16 and F3–3 expressing Adenoviral vectors. SBA titres at 16 weeks post vaccination for Ad-P1.7,16 and Ad-F3–3 expressing adenoviral vaccines measured against target strain 44/76-SL, using baby rabbit complement and pooled mouse sera (*N* = 4 to 8 as described in Figs. 2 and 3).

PorA P1.7,16 vaccines	rSBA titre	FetA F3–3 vaccines	rSBA titre
Empty Vector	< 1:4	Ad-F3–3	< 1:4
Ad-P1.7,16	< 1:4	Ad-FliC-F3–3	< 1:4
Ad-Cytosolic-P1.7,16	< 1:4	Naive	< 1:4
Ad-FliC-VR1.7	< 1:4		
Ad-FliC-VR1.7-C	< 1:4		
Ad-FliC-VR1.7-IMX313	< 1:4		
Anti-1.7 mAb control	1:800		
Anti-1.16 mAb control	1:6400		

**Table 2:** Serum bactericidal responses in mice after immunisation with Ad-P1.7,16 or OMVs. SBA titres at two (week 2) and 6 weeks (week 14) post prime and boost immunisations respectively for Ad-P1.7,16 and OMV (44/76 FetA_*on*_PorA_*on*_ *)* SBA titres measured against target strain 44/76-SL performed using human complement and pooled mouse serum (*N* = 5, as described in Fig. 4A). ‘-’ indicates no boost.

Prime		Boost	
Prime vaccine	hSBA titre Week 2	Boost vaccine	hSBA titre Week 14
Ad-P1.7,16	1:4	OMV	1:128
OMV	1:64	OMV	1:1024
Ad-P1.7,16	1:4	-	< 1:4
Naive	1:4	-	< 1:4

**Table 3:** Serum bactericidal responses in mice after immunisation with Ad-F3–3, Ad-FliC-VR3–3 or OMVs. rSBA titres 6 weeks after prime and 6 weeks after boost (week 14) for Ad-F3–3, Ad-FliC-VR3–3 and OMV (44/76 FetA_*on*_PorA_*off*_*)*. SBA titres measured against target strain 44/76-SL performed using pooled mouse sera (*N* = 8, as described in Fig. 4B). ‘-’ indicates no boost.

F3–3 Vaccines			
Prime vaccine	Week 6 rSBA	Boost vaccine	Week 14 rSBA
Naive	<1:4	–	–
Ad-F3–3	<1:4	OMV-44/76 FetA_on_PorA_off_	<1:4
Ad-FliC-VR3–3	<1:4	OMV-44/76 FetA_on_PorA_off_	<1:4
OMV-44/76 FetA_on_PorA_off_	<1:4	OMV-44/76 FetA_on_PorA_off_	1:32

**Table 4:** hSBA titres in response to Ad-P1.7–2,4 and OMV vaccines. Individual mouse sera were tested using human complement. GMT with 95% CI for each group of mice for each time-point are shown. *N* = 8 BALB/c mice. ^∗^ indicates a significant difference between the OMV prime (week 6) and OMV boost (week 14) sera *P* = .018 by Mann Whitney T test. ‘-’ indicates no boost (column 3) and not done (columns 2 and 4), as no SBA was expected for these test groups at these points.

P1.7–2,4 Vaccines			
Prime vaccine	Week 6 hSBA	Boost vaccine	Week 14 hSBA
Naive	3.0 (1.0, 9.6)	–	–
Ad-P1.7–2,4	–	OMV- NZ98/254	58.7 (12.3,280.0)
OMV- NZ98/254	17.7 (5.1, 61.7)	OMV- NZ98/254	78.0* (48.0, 126.7)
