# Supplementary material for: Viral vectors expressing group B meningococcal outer membrane proteins induce strong antibody responses but fail to induce functional bactericidal activity
Source: J Infect. Author manuscript; Available in PMC 2024 Sep 26. (PMC7616632; doi:10.1016/j.jinf.2022.02.032)
Supplement: Suppl [file EMS198677-supplement-Suppl.docx]

| **Transgene name** | **Description of Transgene** | **N Terminal** | **C Terminal** |
| --- | --- | --- | --- |
| **P1.7, 16** | Full length PorA from strain 44/76 | RKKL | RHKF |
| **Cytosolic-P1.7, 16** | Full length PorA from strain 44/76 without a tPA secretory signal so PorA is not secreted by mammalian cells | RKKL | RHKF |
| **FliC-VR1.7** | PorA VR1 (1.7) from 44/76 inserted into a FliC scaffold to aid presentation of the VR loop | AQAA | VTKA |
| **FliC-VR1.7-C** | PorA VR1 (1.7) from 44/76 inserted into a FliC scaffold with cysteine residues to improve conformation of VR loop | AQAA | VTKA |
| **FliC-VR1.16** | PorA VR2 (1.16) from 44/76 inserted into a FliC scaffold to aid presentation of the VR loop | YYTK | TLVP |
| **FliC-VR1.16-C** | PorA VR2 (1.16) from 44/76 inserted into a FliC scaffold with flanking cysteine residues to improve conformation of VR loop | YYTK | TLVP |
| **FliC-VR1.7 + 16** | PorA VR1 (1.7) and VR2 (1.16) from 44/76 inserted into a FliC scaffold* to aid presentation of the VR loops | AQAA | TLVP |
| **FliC-VR1.7 + 16-C** | PorA VR1 (1.7) and VR2 (1.16) from 44/76 inserted into a FliC scaffold* with flanking cysteine residues to improve conformation of VR loops | AQAA | TLVP |
| **FliC-VR1.7-IMX313** | PorA VR1 (1.7) from 44/76 inserted into a FliC scaffold to aid presentation of the VR loop with an additional IMX313 tag for multimerisation | AQAA | VTKA |
| **F3-3** | Full length FetA from strain 44/76 | RTNI | NYKF |
| **FliC-VR3-3** | Loop 5 of FetA from 44/76 76 inserted into a FliC scaffold to aid presentation of the VR loop | SKFS | YKLS |
| **P1.7-2, 4** | Full length PorA from strain NZ98/254 | RKKL | RHKF |
| **FliC-VR1.4-C** | PorA VR2 (1.4) from NZ98/254 inserted into a FliC scaffold with cysteine residues to improve conformation of VR loop | HVVV | THVP |

**Table S1.** Description of transgene designs. N and C terminal sequences are for the antigen without restriction sites, tPA leader sequence, V5 tag, polylinkers or cysteine residues.

* Contained a flexible polylinker sequence SGMPGSGPAY between VRs

**
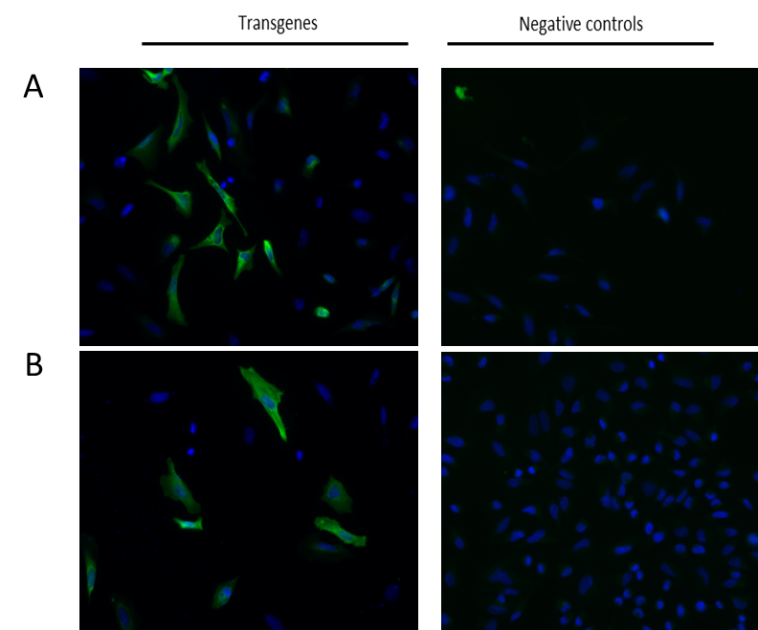
**

**Fig. S1. Detection of simultaneous expression of VR1.7 and VR1.16.** HeLa cells were transfected with plasmids coding for the transgene PorAVR1.7 + 16-C detected with anti-P1.7 mAb (A) or anti-P1.16 mAb (B) (Green-secondary antibody). Negative controls (right image of each panel) were stained identically to the test cells (left image each panel) but were transfected with empty plasmids. Nuclei were counterstained using DAPI (Blue).


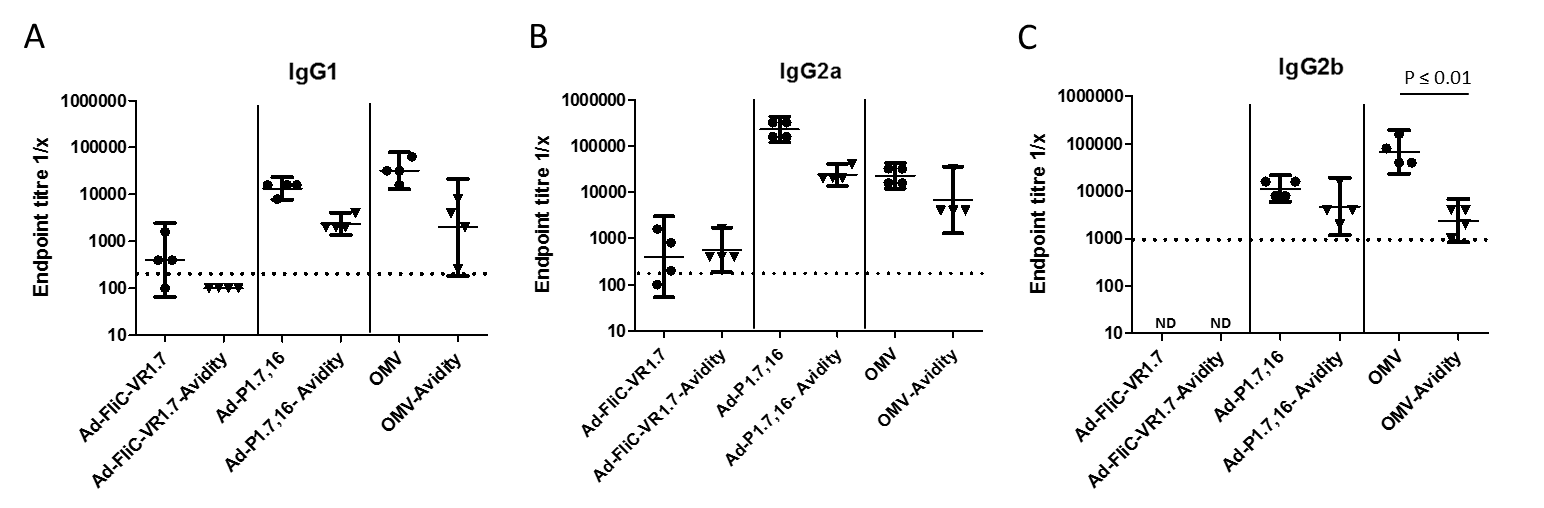


**Fig. S2. IgG subclass ELISA with relative avidity in the presence of chaotropic salts for Ad-P1.7,16 and OMV vaccines.** Sera from groups of 4 mice immunised with a single dose of 10^9^ IU of Ad-P1.7,16 or Ad-FliC-VR1.7 or two doses of OMV (5µg, delivered 8 weeks apart) were used to perform ELISA against rP1.7,16 with (Avidity) and without the addition of 1M sodium thiocyanate. Different secondary antibodies were used to specifically detect IgG1, IgG2a or IgG2b antibodies (A,B and C respectively). The negative cut-off is represented by a dashed line. The horizontal bars indicate the geometric mean with 95% confidence intervals.
